# Full Genome Characterization of the First Oropouche Virus Isolate Imported in Europe from Cuba

**DOI:** 10.3390/v16101586

**Published:** 2024-10-09

**Authors:** Michela Deiana, Simone Malagò, Antonio Mori, Silvia Accordini, Andrea Matucci, Rebeca Passarelli Mantovani, Natasha Gianesini, Ralph Huits, Chiara Piubelli, Federico Giovanni Gobbi, Maria Rosaria Capobianchi, Concetta Castilletti

**Affiliations:** 1 Department of Infectious-Tropical Diseases and Microbiology, IRCCS Sacro Cuore Don Calabria Hospital, Negrar di Valpolicella, 37024 Verona, Italy; michela.deiana@sacrocuore.it (M.D.); simone.malago@sacrocuore.it (S.M.); antonio.mori@sacrocuore.it (A.M.); silvia.accordini@sacrocuore.it (S.A.); andrea.matucci@sacrocuore.it (A.M.); rebeca.passarellimantovani@sacrocuore.it (R.P.M.); natasha.gianesini@sacrocuore.it (N.G.); ralph.huits@sacrocuore.it (R.H.); federico.gobbi@sacrocuore.it (F.G.G.); mrcapobianchi@gmail.com (M.R.C.); concetta.castilletti@sacrocuore.it (C.C.); 2 PhD National Programme in One Health Approaches to Infectious Diseases and Life Science Research, Department of Public Health, Experimental and Forensic Medicine, University of Pavia, 27100 Pavia, Italy; 3 Department of Clinical and Experimental Sciences, University of Brescia, 25121 Brescia, Italy

**Keywords:** Oropouche fever, Oropouche virus, phylogenetic analysis, arbovirus, bunyavirus, emerging infectious diseases

## Abstract

On 27 May 2024, the Cuban Ministry of Health reported the first outbreak of Oropouche fever on the island. The etiologic agent, Oropouche virus (OROV), is a poorly understood arbovirus that has been known since the 1960s and represents a public health burden in Latin America. We report the whole-genome characterization of the first European OROV isolate from a returning traveler from Cuba with Oropouche fever-like symptoms. The isolate was obtained from the patient’s serum; whole-genome sequencing was performed by next-generation sequencing, followed by phylogenetic analysis and genetic variability studies. The analysis showed that the most closely related sequence was from the French Guiana 2020 outbreak. Interestingly, our isolate is a reassortant virus, included in a highly supported monophyletic clade containing recent OROV cases (Brazil 2015–Colombia 2021), separated from the other four previously known genotypes. More deeply, it was found to be included in a distinct branch containing the sequences of the Brazil 2022–2024 outbreak. The reassortment event involved the S and L segments, which have high similarity with sequences belonging to a new cluster (here defined as OROV_SCDC_2024), while the M segment shows high similarity with older sequences. These results likely describe the viral strain responsible for the current outbreak in Cuba, which may also reflect the ongoing outbreak in Latin America. Further studies are needed to understand how OROV evolves towards traits that facilitate its spread and adaptation outside its original basin, and to track its spread and evolution in the European continent.

## 1. Introduction

On 27 May 2024, the Cuban Ministry of Health reported the first outbreak of Oropouche fever on the island [1]. The etiologic agent of Oropouche fever is a re-emerging and poorly understood pathogen arbovirus: *Oropouche virus* (OROV). The preferred vertebrate hosts for this zoonotic virus have not been clearly established, but include non-human primates, wild birds, and pale-throated sloths. OROV has been isolated from the sylvatic mosquito species *Aedes serratus* and *Coquillettidia venezuelensis*. However, the main vectors maintaining OROV in urban transmission cycles are the Cerotopogonidae genus of biting midges (e.g., *C. paraensis*) and *Culex quinquefasciatus* mosquitoes [2], rather than the anthropophilic Aedes mosquitoes.

Since OROV was first identified in 1955 in Trinidad and Tobago, more than 30 human outbreaks have been reported in several countries in Latin America, thus OROV is one of the most important emerging arboviral threats in this region The largest documented OROV outbreak was reported in Brazil in the late 1970s [3], but the true burden of OROV-induced disease remains unknown because the incidence in the human population has not been assessed due to limited or no systematic surveillance. In late 2023, during surveillance for a major dengue outbreak, a significant increase in Oropouche fever cases was observed in Latin America. As of 16 July 2024, the Pan American Health Organization (PAHO) reported more than 7688 confirmed cases of Oropouche in five countries in the Region of the Americas: the Plurinational State of Bolivia, Brazil, Colombia, Cuba, and Peru [4]. Cases of Oropouche fever have been reported in areas and countries where no autochthonous cases had previously been reported. At the same time, cases were detected for the first time in Cuba, involving 13 of the 15 provinces, in a very different ecological context from the Amazon region [5].

The spectrum of clinical manifestations associated with OROV infection is not fully understood, and very little is known about its distribution and ecology. The incidence of OROV is likely to be underreported, because its symptomatology can be easily confused with other febrile illnesses (e.g., dengue fever and leptospirosis) and specific testing for the virus is still uncommon. Recent reports have raised suspicions that OROV may be vertically transmitted, causing severe pregnancy outcomes. These reports have prompted warnings for increased attention and surveillance of OROV infections by international health agencies [4].

OROV belongs to the Simbu serogroup of the Orthobunyavirus genus of the Peribunyaviridae family. The genome is organized into three single-stranded, negative-sense RNA segments, named according to their molecular size: small (S), medium (M), and large (L). The S segment encodes a nucleocapsid and a non-structural protein (NS) with overlapping open reading frames (ORFs). The M segment encodes two glycoproteins (Gc and Gn) and a non-structural protein (NSm), while the L segment encodes RNA-dependent RNA polymerase (RdRp). Genomic viral RNAs (vRNAs) are neither polyadenylated nor modified at the 5′ end; viral mRNAs are also not polyadenylated, but retain a 5′-methylated cap derived from host mRNA via “cap snatching” mediated by the endonuclease function of RdRp [6].

The genetic exchange mechanism in multi-segmented viruses, known as reassortment, is a common phenomenon observed frequently in nature [7]. This process plays a critical role in viral evolution, leading to genetic diversity. Reassortment occurs when two or more viruses co-infect the same cell and during the replication exchange entire genome segments. The resulting viral progeny acquire novel combinations of genetic material, which can lead to significant phenotypic alterations, such as changes in virulence or immune evasion [8]. A wide range of viruses are reported to be able to reassort; for example, influenza viruses frequently undergo reassortment, contributing to the periodic emergence of pandemic strains, including the 2009 H1N1 [9,10], as well as zoonotic viruses and Bunyavirales. Reassortment of the three segments have been described for OROV and can occur through co-infection with different OROV strains or with other Simbu serogroup viruses [11], favoring a viral evolution which can promote viral spread by altering vector competence or virulence [12]. In particular, the recent outbreak in Brazil involves an OROV strain whose genomic segments appear to be derived from different OROV ancestors. This reassortment event has been cited as an event potentially responsible for altered virus characteristics that promote aggressive virus spread [2]. To date, phylogenetic analysis of OROV based on the few complete coding sequences of the S segment available in GenBank has identified four genotypes [13].

Epidemiologic surveillance should be performed to evaluate the occurrence and impact of potentially emerging novel genotypes or reassortants, with implications for molecular diagnostics, the design of potential vaccines, and the understanding of the features underlying viral pathogenesis.

Herein, we report the molecular characterization, based on the complete genome sequence, of the first viral isolate ever reported in Europe outside Latin America, from a patient with Oropouche fever returning to Italy from Cuba in late May 2024. The characteristics of the virus described here are likely to be representative of the characteristics of the strain responsible for the current outbreak in Cuba.

## 2. Materials and Methods

### 2.1. Virus Isolation

A virus isolate was obtained from the serum sample of a patient returning from Cuba and admitted to the IRCCS Sacro Cuore Don Calabria (SCDC) Hospital in Negrar di Valpolicella (Verona, Veneto Region, Italy), where the OROV infection was diagnosed [14]. After collection, the serum was stored at −80 °C and never thawed before inoculation into cell cultures for virus isolation under BSL-3 conditions at the SCDC. The serum sample was diluted 1:10 in MEM (Life Technologies Europe, Bleiswijk, The Netherlands) and then inoculated onto Vero E6 cells (ATCC, Manassas, VA, USA). After 1 h of adsorption, the inoculum was removed and replaced with MEM containing 2% FBS. Cytopathic effect (CPE) was observed by light microscopy after 5 days. The light microscope observed CPE seemed to be common to that of other RNA viruses, with cells rapidly shrink, become dense, and detach from the culture substrate. Cells were removed by low speed centrifugation, the supernatant containing the virus was aliquoted and stored at −80 °C. Subsequent passages were also performed. The virus isolate was designated OROV_IRCCS-SCDC_1/2024_.

### 2.2. RNA Extraction, Library Preparation and Sequencing

Viral RNA was extracted from 140 μL of the supernatant harvested from the first CPE-positive culture and eluted in 60 μL using the QIAamp Viral RNA Mini Kit (Qiagen, Hilden, Germany), according to the manufacturer’s instructions. RNA concentration and integrity were determined before library preparation using the Qubit RNA HS assay kit (Invitrogen, Thermo Fisher Scientific, Inc., Waltham, MA, USA) and the High Sensitivity RNA ScreenTape on the 4200 TapeStation System (Agilent Technologies Inc., Santa Clara, CA, USA). Extracted RNA was used to generate complete genome sequences using a metagenomic approach. Briefly, libraries were constructed using 300 ng of extracted RNA, using the Stranded Total RNA Prep with Ribo-Zero Plus (Illumina Inc., San Diego, CA, USA), according to the manufacturer’s protocol. The library profiles were assessed with a D1000 ScreenTape kit on the 4200 TapeStation System (Agilent Technologies Inc.). Sequencing was performed using the NextSeq1000 (Illumina Inc.), loading 650 pM of library in a P2 flowcell, using 150 base-length read chemistry in a paired-end mode.

### 2.3. Bioinformatic Analysis

#### 2.3.1. Reads Processing, Assembly and Consensus

Base-calling was performed by Illumina’s Dragen RNA tool v4.2.7. Kraken2 (https://github.com/DerrickWood/kraken2, accessed on 1 July 2024) was used to assign taxonomy to the reads with default parameters, and KrakenTools v1.2 (https://github.com/jenniferlu717/KrakenTools, accessed on 1 July 2024) to exclude human reads from the total. Human-filtered reads were then aligned against the three Oropouche complete segments (KP052852.1, KP052851.1, KP052850.1) using Bwa-mem2 v2.2.1 (https://github.com/bwa-mem2/bwa-mem2, accessed on 1 July 2024). Reads were assembled into contigs via SPAdes v3.15.5 (https://github.com/ablab/spades, accessed on 1 July 2024).

Obtained reads were aligned against the KP052852.1, KP052851.1, KP052850.1 sequences exploiting Bwa-mem2 v2.2.1. Alignment metrics were collected via samtools coverage v1.18 (https://github.com/samtools/samtools, accessed on 2 July 2024). Consensus sequences for each segment were obtained using contigs via iVar consensus v1.4.2 (https://github.com/andersen-lab/ivar, accessed on 2 July 2024).

#### 2.3.2. Variant Calling and Annotation

Variant calling was performed using BCFtools v1.18 (https://github.com/samtools/bcftools, last accessed on 2 July 2024). Variant annotation was carried out via SnpEff v5.2c (https://pcingola.github.io/SnpEff/#snpeff, last accessed on 29 July 2024).

The complete sequences of the BeAn19991 strain released in 2015 (KP052852.1, KP052851.1, KP052850.1) was used as reference sequence for the reconstruction of the three genome segments [15].

#### 2.3.3. GenBank Data Download

A total of 197 complete sequences (i.e., 67 for segment M, 67 for segment L and 63 for segment S) were downloaded from the GenBank repository (https://www.ncbi.nlm.nih.gov/genbank/, accessed on 3 July 2024), and used for the comparison with the assembled sequence [16].

The set of retrieved sequences comprised 193 OROV, 1 Madre de Dios (MDDV), 2 Perdoes (PDEV) and 1 Iquitos (IQTV) sequences the isolate here described.

#### 2.3.4. Phylogenetic Analysis and Genetic Variability

To investigate the phylogenetic evolution, Maximum Likelihood (ML) phylogenetic tree was reconstructed using a dataset including our sample and GenBank complete S sequences, for a total of 64 records. The multiple alignment was performed with MAFFT v7.52. ML phylogeny was inferred using IQ-TREE v2.2.2.7. The tree-files were visualized using iTOL v6.9.1 (http://itol.embl.de, accessed on 5 July 2024). Genetic divergence of S segments among sequences was determined using the MEGA11 software and genotypes assigned according to Vasconcelos et al. [13]. Trees for M and L segments were obtained with the same methods and genotypes were assigned based on their S counterpart, when present.

To investigate potential reassortment events with other members of Simbu serogroup, the multiple sequence alignments of the three collated segments (S + M + L) were scanned using the suite of methods implemented in Recombination Detection Program (RDP4) [17]. The reassortment is indicated by the presence of significative breakpoints detected in pairwise collated sequences alignment.

## 3. Results

### 3.1. Quality Control (QC) Analyses, Read Alignment and Consensus Generation

OROV whole-genome sequences were obtained with average depths of coverage of 606× for the S, 991× for the M, and 246× for the L segments, as detailed in Table 1. Consensus sequences were deposited in GenBank with the following identification references: strain name: OROV_IRCCS-SCDC_1/2024_; S segment ID: PP952117; M segment ID: PP952118; and L segment ID: PP952119.

### 3.2. Variant Calling and Annotation

The consensus sequence was obtained using the KP052852.1, KP052851.1, KP052850.1 OROV complete sequences for the 3 segments, to identify nucleotide mutations, substitutions, insertions and deletions. Variant analysis identified some Single Nucleotide Variants (SNVs) and insertions and deletions (InDel) as shown in Table 2. Variants of the different genes were evaluated using the SNpEff tool for annotation and effect prediction. Most of the changes were synonymous mutations, and none of the variants identified in the analysis were predicted to have a relevant effect on the corresponding gene functions.

### 3.3. Phylogenetic Analysis and Genetic Variability

The evolution and genotype classification model described in the study by Vasconcelos and colleagues, focused on OROV and the related Simbu serogroup viruses, was used to characterize OROV_IRCCS-SCDC_1/2024_ [13].

**Figure 1:**
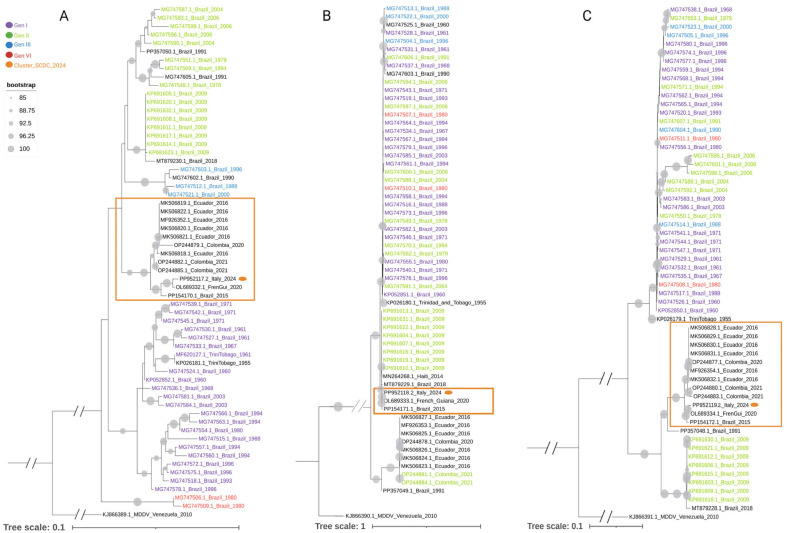
Phylogenetic analysis of OROV sequences of S (panel (**A**); n = 60), M (panel (**B**); n = 64) and L segment (panel (**C**); n = 64). ML tree based is rooted on the MDDV sequence used as outgroup. The scale bar represents the nucleotide distance, and the gray circles represents the bootstrap values (>85). The sequences already assigned to the 4 established genotypes according to the literature [13] are highlighted with different character colors. Sequences not yet included into a known genotype are in black characters. The sequence of the OROV_IRCCS-SCDC_1/2024_ here described for the first time (S segment ID: PP952117.2; M segment ID: PP952118.2; and L segment ID: PP952119.2) are highlighted with an orange dot. The distinct cluster OROV_SCDC_2024 is squared with an orange rectangle.

The phylogenetic analysis was performed on 64 full-length S segment (951 bp) sequences; among these, sequences with known genotype were used to trace the genotype distribution in the phylogenetic tree [12]. The results, shown in Figure 1, indicate that across all the three genomic segments the sequence of OROV_IRCCS-SCDC_1/2024_ was included in a highly supported (PP > 0.90) monophyletic clade (OROV_SCDC_2024) and that the most closely related sequences were those from a patient in French Guiana during the 2020 outbreak.

Interestingly, the S segment sequence of OROV_IRCCS-SCDC_1/2024_ clustered with sequences from cases identified after 2015 (Brazil 2015–Colombia 2021), whose genotype attribution has not been formalized yet, forming a well-supported branch (bootstrap (>90) that was clearly separated from the clusters of the other four previously known genotypes.

The genetic distance of the OROV_SCDC_2024 cluster towards known genotypes was evaluated for the full-length S segment [13]. The results, reported in Table 3, indicate that the mean genetic distance vs. genotypes I to IV ranged between 3.4% and 6% (Table 3); mean genetic distance of the new OROV_SCDC_2024 cluster vs. all the genotypes was 6.4%.

**Figure 2:**
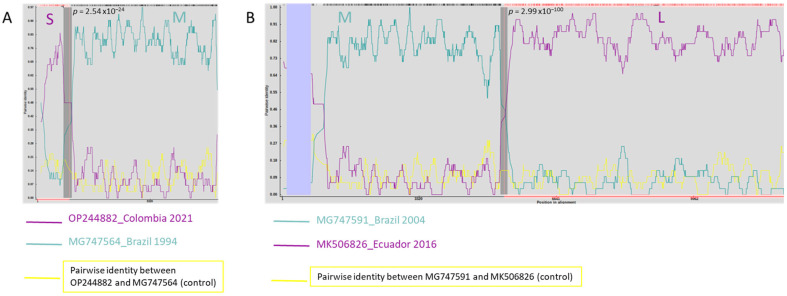
RDP output for OROV_IRCCS-SCDC_1/2024_. The three segments’ collated sequences (S + M + L) were imputed in the RDP tool for the reassortment analysis. Interface between segments S and M (panel (**A**)) and interface between segment M and L (panel (**B**)) are reported. The sequences resulting with the highest pairwise identity with OROV_IRCCS-SCDC_1/2024_ for S, M and L regions are reported (green and purple lines). The reassortment is indicated by the presence of significative breakpoints detected in pairwise sequence alignment. Grey areas highlight the breakpoints of the collated sequences with their associated *p* values.

Recombination Detection Program (RDP4) identified significant (i.e., *p* ≤ 0.05) reassortment events in OROV_IRCCS-SCDC_1/2024_, seemingly within-species (Figure 2). In particular, the S and L segments have high similarity with sequences belonging to the new OROV_SCDC_2024 cluster different from the 4 known genotypes (respectively, the OP244882, detected in Colombia in 2021 and the MK506826, detected in Ecuador in 2016, Figure 2), while the M segment has high similarity with MG747564 (detected in Brazil in 1994, Figure 2) and MG747591 (detected in Brazil in 2004, Figure 2). The viruses of the latter two sequences were classified, based on their S sequence, as Genotype I and II respectively, but their M segments show 99.41% of sequence identity (Clustal W v2.1 [18]).

A recent analysis of OROV strains circulating in the Brazilian outbreak, which has been ongoing since 2022, was based on sequences shorter than those analyzed in the present study [19,20,21]. This study showed that the sequences from the Brazilian outbreak form a distinct clade from all the previously known sequences, and the authors suggested that the OROV strain responsible for the current outbreak in Brazil inherited its genetic segments from different OROV progenitors. This hypothesis was further confirmed by additional genetic analyses performed on cases of the same outbreak [22].

Considering this recent information, we performed the additional analysis presented in Supplementary Figures S1–S3. Briefly, we compose phylogenetic trees including also incomplete sequences, after having trimmed the OROV_IRCCS-SCDC_1/2024_ sequence and those used in Figure 1, to obtain overlapping partial sequences for all the three segments. The phylogenetic trees of all segments confirmed that the Brazil 2022–2024 sequences form a distinct branch, including OROV_IRCCS-SCDC_1/2024_ sequence. In addition, the closest branch includes sequences from recent cases outside Brazil (i.e., from Ecuador and Colombia), that were not included in the analysis by Naveca and colleagues [21].

## 4. Discussion

Despite a long history of outbreaks, the number of full-length OROV genomes obtained since its first identification in 1960 is small (n = 64, including OROV_IRCCS-SCDC_1/2024_) making it difficult to understand the evolutionary and spread dynamics of this arbovirus [23]. Thus, there is a critical need to expand diagnostic and sequencing efforts to improve our understanding of the molecular evolution, transmission dynamics, and disease burden of OROV and, in the current changing ecological and epidemiological landscape, its potential spread outside of Latin America [24].

In this study we performed the molecular characterization of the first OROV isolate, named OROV_IRCCS-SCDC_1/2024_, obtained outside of its original distribution area, in a patient travelling from Cuba back to Italy. We obtained high-quality full-length sequences of all 3 genomic segments that are now available in GeneBank (ID: PP952117, PP952118, PP952119).

By sequence analysis, we detected several single nucleotide variants (SNVs) in OROV_IRCCS-SCDC_1/2024_ compared to the reference sequence (the sequence of the BeAn19991 strain, isolated in 1960 in Brazil). and none of them was predicted to have a relevant effect on the gene in which it was located.

Phylogenetic analysis revealed that OROV_IRCCS-SCDC_1/2024_ (PP952117) was most closely related to a strain isolated in French Guiana (OL689332) during the 2020 outbreak [25]. This similarity was previously observed for sequences detected in Western Amazon in 2022–2023 [20]. In our analysis, PP952117 and OL689332, together with a sequence detected in 2015 in Brazil (PP154170), appeared to belong to an independent, well-supported cluster [bootstrap (>90)] distinct from any described genotype (Figure 1). This cluster, named OROV_SCDC_2024, also contains sequences from outbreaks in Ecuador (2016) and Colombia (2020) [26,27].

The phylogenetic tree based on L sequences fairly corresponds to what observed in the tree based on S sequence, where the OROV_SCDC_2024 cluster is well separated from sequences belonging to known genotypes. Focusing on the M sequence, the separation is less evident and the cluster span in closed but different branches of the phylogenetic tree.

As of today, four OROV genotypes have been described based on genetic divergency of S fragment, defining the mean genetic distance among genotypes at ~5% [13,28]. In our analyses, the mean genetic distance of the OROV_SCDC_2024 cluster vs. genotypes I to IV ranged between 3.4% and 6% (Table 3), and mean genetic distance vs. all the known genotypes was 6.4%, consistent with the presence of a new genotype, as previously described [13].

In the comparison against other OROV complete and concatenated sequences, RDP4 bioinformatic tool identified reassortment events in OROV_IRCCS-SCDC_1/2024_ (i.e., *p* value ≤ 0.05), suggesting within-species reassortment events. The reassortment analysis showed results in line with what observed from the phylogenetic analysis of the 3 segments. In particular, the S and L segments resulted to have high similarity with sequences belonging to the OROV_SCDC_2024 cluster, probably deriving from recent ancestors (originating after 2015). The M segment instead showed high similarity with two older sequences (probably deriving form older ancestors, already detected in 1994). The two older sequences were classified, based on S segment, as Genotype I and II respectively, but with 99.41% of sequence identity in their M segments.

OROV reassortment occurrence has been well described in previous studies [2,12]. Recently, Naveca et al. reported that a novel reassortant virus appeared to be responsible for the large OROV outbreak affecting four states in the Brazilian Amazon region between August 2022 and March 2024 [21]. Further genomic analysis of sampled cases from the same outbreak identified multiple reassortment events, suggesting a high frequency of this phenomenon [22]. The biological characterization of the OROV_IRCCS-SCDC_1/2024_ is ongoing to evaluate a possible higher replication efficiency as observed for the reassortant strain responsible for the 2022–2024 Brazilian outbreak [29], where a ~100-fold higher replication efficiency has been observed compared to the prototype strain BeAn19999, with possible implications in virus spread. Indeed, reassortment, especially in the case of viruses with segmented genomes, represents one of the main mechanisms of evolution [30]. It helps generate genetic diversity and plays an important role in the emergence and spread of new strains with altered virulence, as amply demonstrated in the case of influenza viruses.

Moreover, the phylogenetic characterization of the Brazilian OROV outbreak [21,22] highlighted a new branch with a highly supported (PP > 0.90) monophyletic clade (OROVBR-2015-2024) across all three genomic segments. This branch included the sequence of French Guiana 2020 OROV [21]. We did not include the Brazil 2022–2024 sequences in the phylogenetic trees shown in Figure 1 because, as stated, these trees were built only with complete sequences retrieved from GenBank.

In order to include sequencing from the current Brazilian outbreak, we performed an additional phylogenetic analysis, which also included incomplete sequences, after having trimmed the complete sequences in order to restrict the analysis to matching overlapped segments. These trees (Figures S1–S3 in Supplementary Materials) confirmed the existence of a distinct, unique cluster that included the recent sequences from Brazil highlighted in the study of Naveca et al. [21], and the OROV_IRCCS-SCDC_1/2024_ sequence.

Our data further supports the indications that the ongoing outbreak in Latin America and surrounding territories, is sustained by a virus with distinct genetic characters. The origin of this new strain is inferred to have occurred around 2015, and it involves a reassortment event between different OROV strains, mainly involving the S and the L segments. Implications on vector competence and virus transmission need to be further explored.

## 5. Conclusions

To our knowledge, this is the first OROV whole genome sequence from a virus isolate obtained outside regions with endemic circulation of OROV. This isolate probably reflects the genetic characteristics of the OROV strain that is involved in the ongoing outbreak in Latin America. In particular, it likely represents the strain that is maintaining the current outbreak in Cuba, and it may have characteristics that differ from those observed in previous outbreaks. It is possible that genetic changes that dominate the currently circulating strain are responsible for the upsurge and geographic expansion of OROV outbreaks outside of known endemic areas. The increase of human travel opportunities, together with upcoming mass gathering events in Europe, highlight the need for increased surveillance efforts and clinical awareness [24]. It is necessary to expand the sequencing coverage to better characterize the viral strains that are expanding their geographic distribution. Further studies are needed to assess the potential impact of genetic changes in OROV biological characteristics, to understand if OROV is evolving towards the development of traits that favor its geographic spread, and, more importantly, the adaptation outside of its original basin, including arthropod vector tropism and requirement of amplifying host(s). Additional studies are necessary to fill the knowledge gaps in disease severity and virus transmissibility in susceptible populations, particularly in the light of recent alarming reports that OROV may cause stillbirths and neurological defects in infants infected in the womb [4].

## Figures and Tables

**Table 1:** QC results of sequencing data. For each segment, the columns show: the GenBank ID, the number of sequenced fragments, the number of mapped fragments on OROV reference, the mean depth of coverage and the percentage of target sequences covered at least by 10/30/1000 reads.

	Accession ID	Total Number of Sequenced Fragments	Fragments Mapped on OROV (*n*)	Coverage (%)	Mean Depth Coverage (X)	10× (%)	30× (%)	1000× (%)
S	PP952117	88,041,642	5632	99.27	606	99.16	98.95	19.41
M	PP952118	88,041,642	34,481	99.73	991	99.15	92.26	38.15
L	PP952119	88,041,642	15,899	99.31	246	97.03	93.03	4.27

**Table 2:** Results of assembled segments. For each sequence the columns show the GenBank accession id, the total length of the genome assembled with spades, and the total number of identified SNVs and InDels by bcftools. The number of synonymous and missense variants by snpeff analysis is also reported.

	Accession ID	Assembly Length	Coverage (%)	# SNVs	# Dels.	# Ins.	# Missense	# Synonymous
S	PP952117	951	99.27	38	1	0	2	34
M	PP952118	4400	99.73	165	0	0	23	138
L	PP952119	7700	99.32	727	0	1	54	637

**Table 3:** Percentage of genetic divergence (mean% ± the standard error) between OROV genotypes and cluster OROV_SCDC_2024. Genetic divergence was evaluated on full-length S sequences.

	Gen I	Gen II	Gen III	Gen IV	Mean Intragroup Divergence
**Gen I**	-				0.024
**Gen II**	5.0 ± 0.6	-			0.018
**Gen III**	5.9 ± 0.7	3.9 ± 0.6	-		0.007
**Gen IV**	6.2 ± 0.8	5.6 ± 0.7	6.4 ± 0.9	-	0.004
**OROV_SCDC_2024**	5.1 ± 0.6	3.4 ± 0.5	4.8 ± 0.7	6.0 ± 0.8	0.010

## Data Availability

All genomic data referring the produced sequences have been uploaded on GeneBank (NCBI) and are publicly available.

## Supplementary Materials

The following supporting information can be downloaded at: https://www.mdpi.com/article/10.3390/v16101586/s1, Figure S1: Phylogenetic tree of S segment. Labels displaying only the accession ID indicate OROV sequences obtained in Brazil from 2022 to 2024 [21]. Figure S2: Phylogenetic tree of M segment. Labels displaying only the accession ID indicate OROV sequences obtained in Brazil from 2022 to 2024 [21]. Figure S3: Phylogenetic tree of L segment. Labels displaying only the accession ID indicate OROV sequences obtained in Brazil from 2022 to 2024 [21].
