# Supplementary figures and images for: Full Genome Characterization of the First Oropouche Virus Isolate Imported in Europe from Cuba
Source: Viruses. 2024 Oct 9;16(10):1586. doi: 10.3390/v16101586 (PMC11512199; doi:10.3390/v16101586)

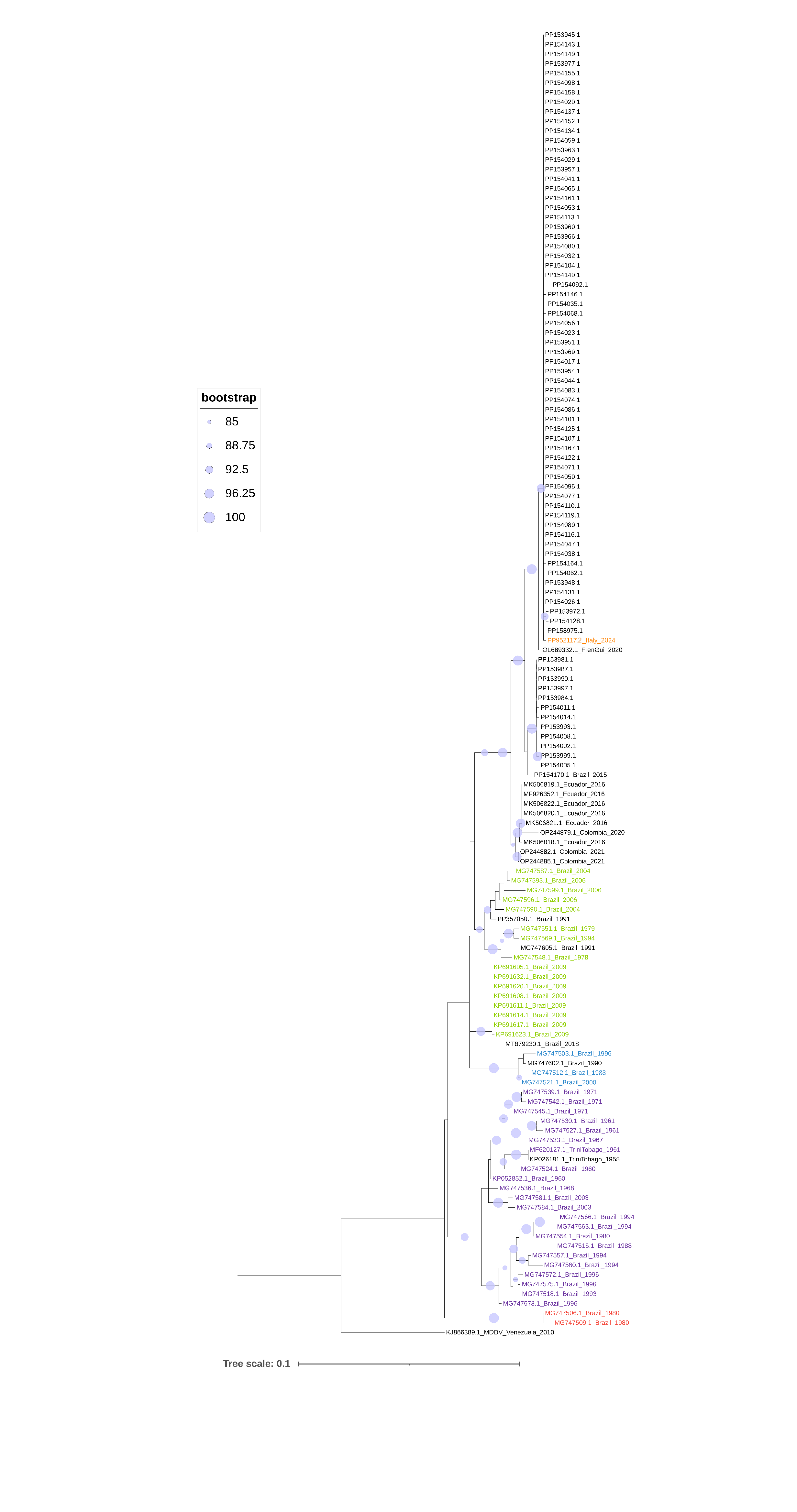

Supplement: Supplementary file 1 [file viruses-16-01586-s001.zip › OROV_Trees_supplementary/1 - suppl Segment S.png]

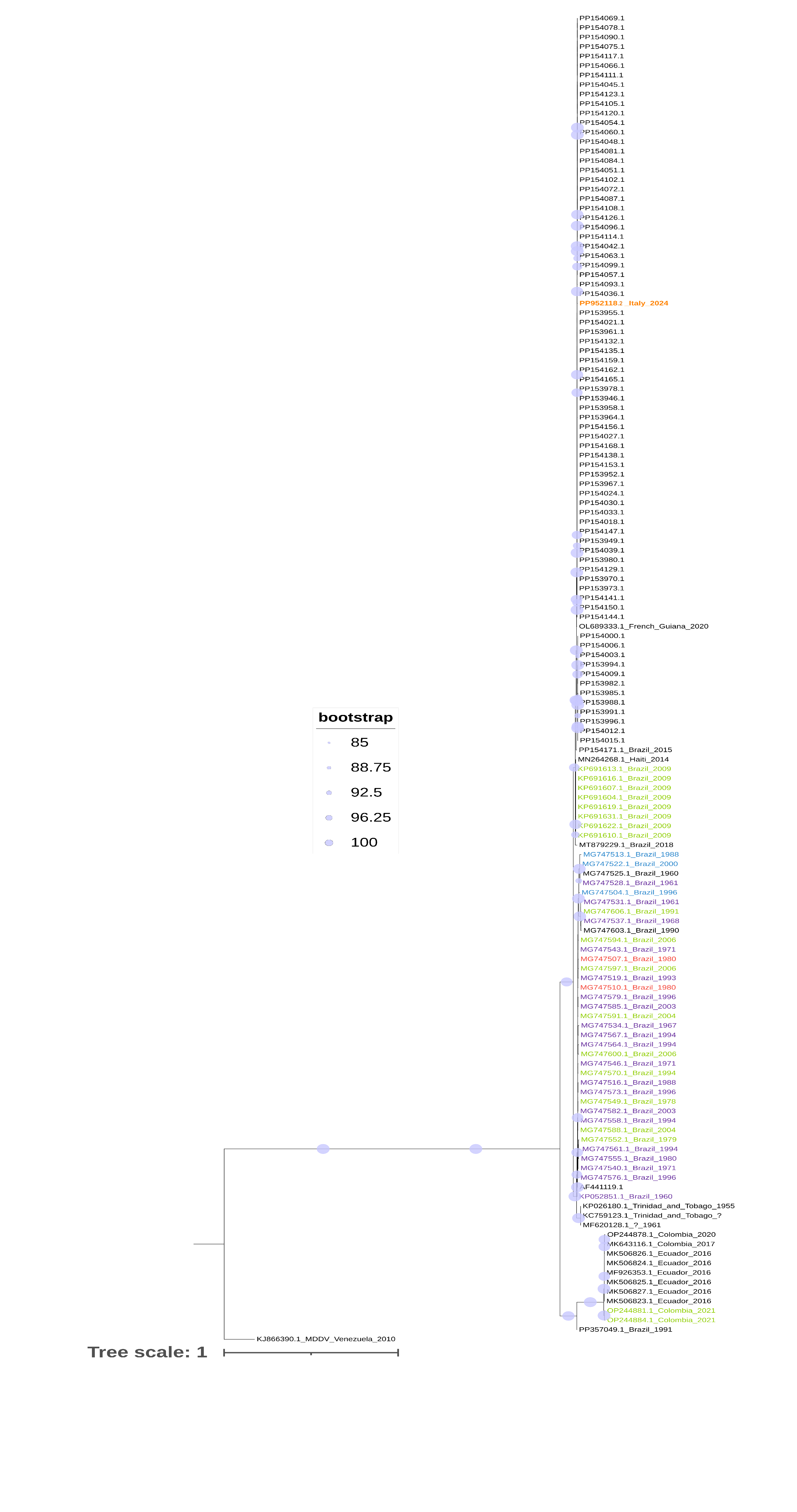

Supplement: Supplementary file 1 [file viruses-16-01586-s001.zip › OROV_Trees_supplementary/2 - Suppl Segment M.png]

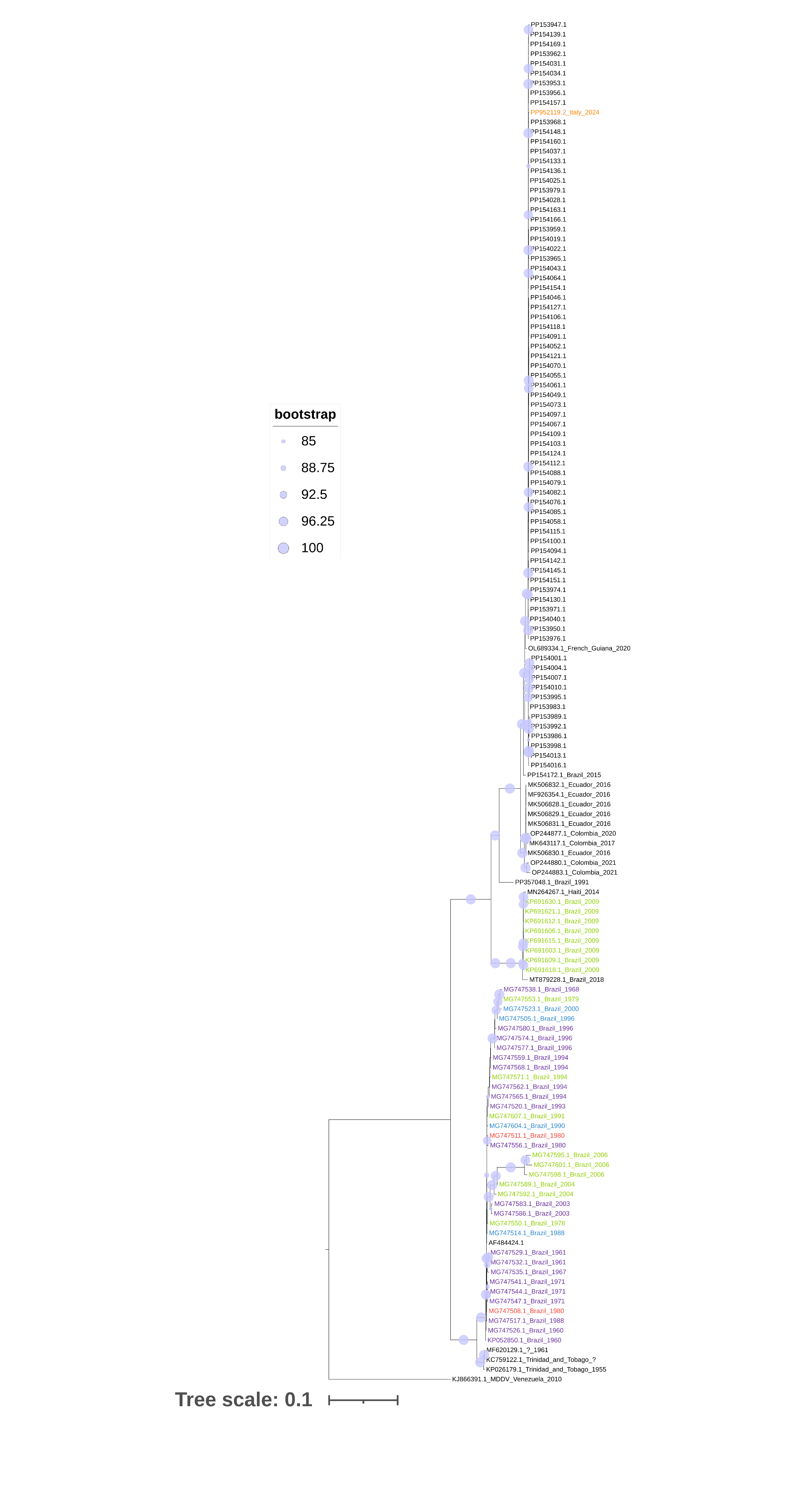

Supplement: Supplementary file 1 [file viruses-16-01586-s001.zip › OROV_Trees_supplementary/3 - Suppl Segment L.png]
